# Adaptive human behavior in epidemics: the impact of risk misperception on the spread of epidemics

**DOI:** 10.21203/rs.3.rs-220733/v1

**Published:** 2021-02-23

**Authors:** Baltazar Espinoza, Madhav Marathe, Samarth Swarup, Mugdha Thakur

**Affiliations:** 1 Biocomplexity Institute and Initiative, Network Systems Science and Advanced Computing Division, University of Virginia, Virginia, USA

## Abstract

Infections produced by pre-symptomatic and asymptomatic (non-symptomatic) individuals have been identified as major drivers of COVID-19 transmission. Non-symptomatic individuals unaware of the infection risk they pose to others, may perceive themselves –and being perceived by others– as not representing risk of infection. Yet many epidemiological models currently in use do not include a behavioral component, and do not address the potential consequences of risk misperception. To study the impact of behavioral adaptations to the perceived infection risk, we use a mathematical model that incorporates individuals’ behavioral decisions based on a projection of the future system’s state over a finite planning horizon. We found that individuals’ risk misperception in the presence of asymptomatic individuals may increase or reduce the final epidemic size. Moreover, under behavioral response the impact of asymptomatic infections is modulated by symptomatic individuals’ behavior. Finally, we found that there is an optimal planning horizon that minimizes the final epidemic size.

## Introduction

Asymptomatic individuals have the potential to affect the course of an epidemic through silent infections. Transmission events in the absence of symptoms have been documented for different diseases, including the ongoing COVID-19 pandemic^1–3^. The emergence of the SARS-CoV-2 virus challenged the scientific community to promptly uncover its pathogenesis and transmission dynamics in order to fight infections and achieve disease containment. The potential transmission of COVID-19 during the pre-symptomatic and asymptomatic stages was recognized relatively quickly^4,5^. Containment efforts involving contact tracing and testing have identified pre-symptomatic and asymptomatic individuals as major drivers of COVID-19 transmission in a number of countries^3,6–10^. However, the impossibility of identifying asymptomatic individuals without testing poses a major challenge for disease containment. Yet most countries only test symptomatic individuals^11^. Ideally, large scale random testing with appropriate test sensitivity is required to characterize the progression routes of infection^12,13^. Without random testing, the role of asymptomatic infections is hard to identify. The problem is made more difficult by the fact that the asymptomatic/symptomatic ratio, as well as the infectiousness potential of COVID-19 asymptomatic individuals is uncertain^14–16^. Studies estimating asymptomatic relative infectiousness report highly variable results, ranging from (0%−40%)^7,10,17^ to (40%−70%)^11,12,18–20^.

Absent testing, infections produced by asymptomatic individuals are difficult to prevent and to track due to the lack of apparent illness and to the fact that asymptomatic individuals are unaware of the infection risk they pose to others. Asymptomatic individuals may perceive themselves –and being perceived by others– as not representing an infection risk, potentially starting infection chains that are not detectable through contact tracing^9^. Yet many epidemiological models currently in use do not include a behavioral component, and do not address the potential consequences of risk misperception.

To get a measure of the risks posed by infectious pre-symptomatic and asymptomatic individuals we consider behavioral responses to perceived risk. Behavioral responses aimed at mitigating disease risk include social distancing by both susceptible and infected individuals, increased use of protective equipment, and better hygiene. Collectively, the suite of behavioral responses taken by individuals have been characterized as a *behavioral immune system* at the population level^21^. Modern mathematical models envision epidemics as complex systems in which behavioral responses, at different scales, both drive and are driven by the disease transmission process. A number of different mathematical modeling frameworks have been used to understand interactions between disease dynamics and behavioral responses,^22–24^.

In this paper we use per-capita contact rates as the mechanism by which disease is transmitted and benefits are obtained. In other words, we assume economic productivity depends on social interactions^25,26^. We apply the framework by Fenichel et. al. in^27–29^, to study how the behavior of infectious exposed and asymptomatic individuals affects the spread of a disease. Specifically, we use the impact of behavioral adaptations to disease risk to understand the effect of asymptomatic infections on the transmission dynamics and the final epidemic size. As in the work by Fenichel et. al., we use a set of ordinary differential equations to model disease progression, and a decentralized Markov decision framework to model the strategic behavior of individuals in different health classes. Behavioral changes are modeled as adjustments in the contact decisions made by individuals seeking to maximize the net benefits offered by contacts with others, where contacts also carry a risk of infection. Specifically, we model the response to disease risk as a trade-off between benefits secured through contact with others, and costs associated with the probability of infection due to contact with others expected to occur over some finite planning horizon. Before the vaccines against COVID-19 infections were widely available, behavioral change took the form of variation in contact rates over the epidemic period. That is, individuals chose their daily contact rates, given their understanding of infection risks, so as to maximize the discounted flow of net benefits over a given planning horizon. The decision process accounts for expectations of future utility, future risk of infection and potential future transitions to alternative health states, based on a projection of the future system’s state over a finite planning horizon.

Understanding of infection risk is assumed to be determined by vulnerability cues^21^. Since most social interactions require immediate evaluation of the infection risk, individuals are biased towards easily observable cues–specifically the presence or absence of symptoms. It follows that there will be at best a weak behavioral response to individuals exhibiting mild or no symptoms. Specifically, we suppose that the impact of non-symptomatic (exposed and asymptomatic) individuals on the transmission dynamics depends on two misperceptions: (i) non-symptomatic individuals are treated as not infectious; (ii) uninfected and non-symptomatic infectious individuals behave as if they are susceptible.

Taking variation in the final epidemic size as a measure of the impact of asymptomatic infections, we consider the net effect of these two misperceptions on the behavior of the non-symptomatic population. The risk-avoiding behavior of non-symptomatic but infectious individuals who perceive themselves to be susceptible, is balanced against the risk-increasing behavior of susceptible individuals who perceive non-symptomatic individuals as non-infectious. Moreover, the impact of the non-symptomatic population’s behavior is conditioned by the behavior of the symptomatic but still socially active population. In the US, as in many other countries, non-pharmaceutical pro-social precautionary measures by infected but socially active individuals are recommended but not mandatory^30,31^. Consequently, it is expected that only a fraction of the infected but socially active population will comply with health authority recommendations. We test the strength of the impact of variations in the behavior of the non-symptomatic population to variations in the proportion of the symptomatic population adopting pro-social behaviors.

We find that under behavioral adaptation an epidemic driven by both symptomatic and asymptomatic cases may produce a greater final epidemic size than the analogous epidemic solely driven by symptomatic cases. Individuals’ risk misperception, by playing a dual role on the behavioral response produced during an epidemic, may ameliorate or exacerbate the epidemic. By contrast, constant contacts models, may find the final epidemic size monotonically decreases when a proportion of infections result in asymptomatic cases.

Moreover, under behavioral response the impact of asymptomatic infections is modulated by symptomatic individuals’ behavior. The lower the symptomatic individuals’ contact rate, the greater the impact of asymptomatic infections on the attack rate.

Finally, we found that there is an optimal planning horizon that minimizes the final epidemic size regardless of the proportion of asymptomatic cases and their relative infectiousness.

## Results

Since the model is not amenable to an analytic solution, we numerically explore the implications of adaptive behavior and risk misperception on the epidemic dynamics and final size. We assume per-contact utility to be independent of health status and use the single peaked utility function $u_{t}={\left(bC_{t}^{h}-{\left(C_{t}^{h}\right)}^{2}\right)}^{v}$, where $C_{t}^{h}$ represents the contact rate of a typical individual with health status *h* at time *t*, and where the maximum number of contacts available per time (*b*) and the utility function shape parameter (*ν*), are fixed over time. Therefore, $u\left(h,C_{t}^{h}\right)$ represents the immediate utility a typical individual in health class *h* obtains by making *C* contacts at time *t*.

Since preferences are single-peaked each individual has a unique most preferred contact rate. Although the utility function is symmetric around the optimal contact rate *C*^∗^ = *b*/2, we restrict behavior adaptations to reductions in the contact rate. In Appendix C we explore the impact of changes in the utility function on the adaptive behavior produced.

In the absence of appropriate behavioral data, we assumed individuals make an average of *b* = 48 contacts per day and that future utility is discounted at the rate of 5% per year (*δ* = 0.99986), and the utility function parameter value is assumed to be *ν* = 0.1,^27,29^. We explore the impact of variations in these parameter values in Appendix C. We calibrate the behavior model letting expression (2) to be consistent with early disease dynamics of the COVID-19 pandemic. Exposed individuals are assumed to exhibit a 5 days latency period (*κ* = 1/5) with a reduced infectiousness of *ρ* = 0.25,^4,35^. Infected individuals recover and cannot infect others on average after 9 days (*γ* = 1/9) of symptoms onset,^36^. For our baseline parameters we assume 50% (*σ* = 0.5) of the infections become asymptomatic with relative infectiousness of *ε* = 0.4^10^, and all symptomatic individuals to be non-compliant (*l* = 1). These baseline parameters with a per-contact likelihood of infection *β* = 0.01324, generate a basic reproductive number of 2.4,^37,38^. The set of parameters used in our numerical experiments, unless otherwise indicated, are collected in Table 1.

We now use model (1) with varying contact rates to study the impact of non-symptomatic individuals’ behavior on the transmission dynamics and final epidemic size where symptomatic individuals are always non-compliant *l* = 1.

### Risk misperception has the potential to increase the final epidemic size

**Figure 1.**
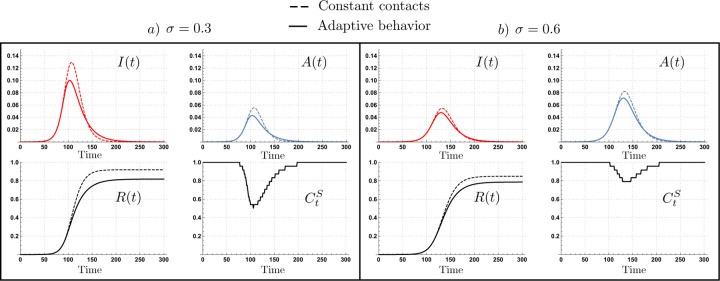
Disease dynamics under adaptive behavior (thick curves) and constant contact rates (dashed curves). The scenarios where 30% (panel a) and 60% (panel b) of cases become asymptomatic show differential behavioral response $\left(C_{t}^{S}\right)$ as a function of the risk perception, impacting the final epidemic size. Parameters *τ* = 14, *ν* = 0.1, *ε* = 0.4,*C*^*I*^ = max*C*_*t*_, *l* = 1 and *ρ* = 0.25.

Figure 1 shows selected simulations for disease dynamics under constant contact rates (dashed curves), and under adaptive behavior (solid curves). It reports two scenarios: in panel (a) 30% (*σ* = 0.3) of all infections are asymptomatic, and in panel (b) 60% (*σ* = 0.6) of all infections are asymptomatic. In panel (a), behavioral adaptation reduces the contact rate of non-symptomatic individuals $\left(C_{t}^{S}\right)$ down to 50%, and in panel (b), there is a weaker behavioral response, reducing the non-symptomatic contact rate to 80%.

**Figure 2.**
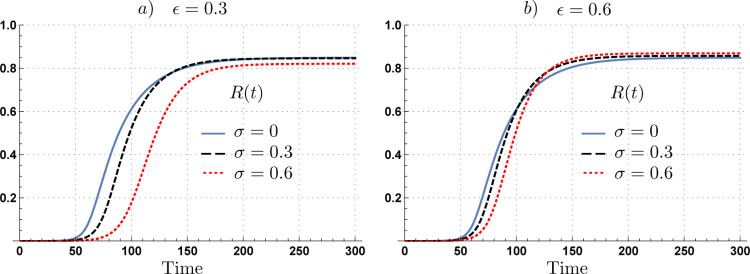
Time evolution of the proportion of recovered population for scenarios with 0%, 30%, and 60% of asymptomatic cases under adaptive behavior model. The impact of the asymptomatic subpopulation on the attack rate depends upon its relative infectiousness. Panel a) shows that increments of the asymptomatic subpopulation having low infectiousness (*ε* = 30%), decreases the attack rate. Panel b), shows that increments on the asymptomatic cases having high infectiousness (*ε* = 60%), increases the attack rate. Parameters *τ* = 14, *ν* = 0.1, *ρ* = 0.3,*C*^*I*^ = max*C*_*t*_, *l* = 1 and *ρ* = 0.25.

While adaptive behavioral responses to disease risk reduce contact rates in both cases, the level of contacts reduction is sensitive to the proportion of infections that are asymptomatic. We now explore the impact of the infectiousness of asymptomatic individuals on the attack rate. Figure 2 shows that the impact of silent infections depends upon the relative infectiousness of asymptomatic individuals. Our simulations show that under low infectiousness of asymptomatic individuals (*ε* = 30%), the attack rate decreases as the proportion of asymptomatic cases increases. However, if asymptomatic individuals are highly infectious, (*ε* = 60%), the attack rate increases as the proportion of asymptomatic cases increases.

**Figure 3.**
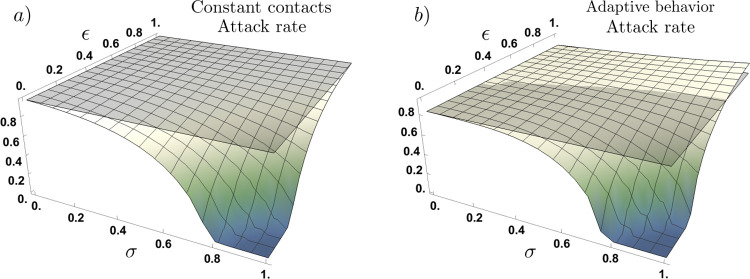
Attack rate under fixed contact rates (panel a) and under adaptive behavior (panel b), as a function of the proportion of asymptomatic infections (*σ*) and their relative infectiousness (*ε*). Panel (a) shows that under constant contact rates, changes on the proportion of asymptomatic infections and their relative infectiousness monotonically decreases the attack rate. Panel (b) shows that under adaptive behavior, there exists scenarios for which the attack rate in the presence of asymptomatic cases overcomes the attack rate of having no asymptomatic cases. In this scenario, the presence of asymptomatic cases increases or decreases the attack rate, relative to their infectiousness. Parameter set *τ* = 14, *ν* = 0.1,*C*^*I*^ = max*C*_*t*_, *l* = 1 and *ρ* = 0.25.

In Figure 3 we explore all the potential scenarios where the attack rate is a function of both the proportion of infections that are asymptomatic (*σ*), and their relative infectiousness (*ε*). Panel a) shows the attack rate for the constant contact rates model, and panel b) shows the attack rate for the adaptive behavior model. We take the case where there are no asymptomatic infections (*σ* = 0) as the baseline scenario (gray plane), for each model. Panel a) shows that under the constant contact rates model, regardless of asymptomatic individuals’ relative infectiousness, an epidemic driven by both symptomatic and asymptomatic cases (*σ* > 0), leads to a lower attack rate than an epidemic solely driven by symptomatic cases. That is, under the constant contact rates model the attack rate attained for all (*σ*, *ε*) scenarios, is lower than the attack rate for the baseline scenario, (*σ* = 0).

Panel b) shows as expected, that the attack rate in the absence of asymptomatic infections under behavioral response is lower than the corresponding one under constant contact rates. Interestingly, under the adaptive behavior model, the attack rate shows a non-monotonic behavior to the presence of asymptomatic infections. For scenarios where asymptomatic individuals’ infectiousness is high (*ε* > 0.6), behavioral response leads to an increased attack rate, relative to the baseline scenario (*σ* = 0). In counterpart, for scenarios where asymptomatic individuals’ infectiousness is low (*ε* < 0.4), behavioral response leads to a reduced attack rate, compared to the baseline scenario. Notice that for intermediate levels of asymptomatic individuals’ infectiousness, the impact of adaptive behavior on the attack rate depends upon the trade-off between the proportion of asymptomatic cases (*σ*) and their relative infectiousness (*ε*).

### Symptomatic individuals’ behavior modulates the impact of asymptomatic infections

Our next set of experiments tested the effect of symptomatic individuals’ activity level. Specifically, we explored the impact of behavioral responses on the attack rate as the contact rate of infected (but still socially active) individuals varies. We found that the attack rate, in the presence of asymptomatic cases, is moderated by the contact rate of symptomatic but still socially active individuals.

**Figure 4.**
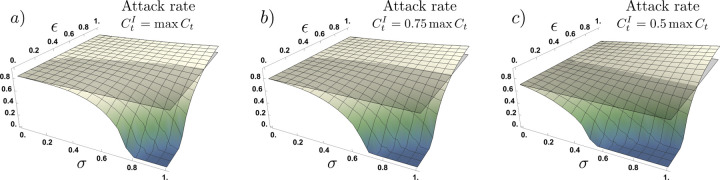
Attack rate as a function of the proportion of asymptomatic infections (*σ*) and their relative infectiousness (*ε*), for different contact rates of symptomatic individuals under adaptive behavior model. In panel a) we assume symptomatic individuals maintain the privately optimal contact rate $\left(C_{t}^{I}=\max C_{t}\right)$, in panel b) we assume symptomatic individuals reduce their contact rate to 75% $\left(C_{t}^{I}=0.75\max C_{t}\right)$, and in panel c) we assume a contact rate reduction to 50% $\left(C_{t}^{I}=0.5\max C_{t}\right)$. Compliance with recommended precautionary measures by infectious individuals moderates the impact of asymptomatic infections. The lower the symptomatic individuals’ contact rate, the greater the impact of asymptomatic infections on the attack rate. Parameters *τ* = 14, *ν* = 0.1, and *ρ* = 0.25.

Figure 4 shows the impact on the attack rate, of the proportion of asymptomatic cases (*σ*) and their relative infectiousness (*ε*), for scenarios where symptomatic infected individuals exhibit contact rates of $C_{t}^{I}=100\%$, 75% and 50%. Our simulations show that, in general, the attack rate of the epidemic decreases as the contact rate of symptomatic individuals falls, an intuitive result. Moreover, Figure 4 shows two effects on the attack rate as symptomatic contact rates decreases: (i) the impact of asymptomatic infections increases as the symptomatic individuals are less socially active, increasing the attack rate over the baseline scenario, (ii) the higher the level of compliance (the reduction in the symptomatic individuals’ contact rate), the lower the levels of asymptomatic cases and relative infectiousness (*σ*, *ε*) at which the attack rate exceeds the baseline scenario (*σ* = 0). In other words, the (*σ*, *ε*) values that lead to an increased final epidemic size over the base case decrease as symptomatic compliance increases. Furthermore, for the scenarios at which the presence of asymptomatic individuals exceeds the baseline scenario (no asymptomatic cases), the impact on the attack rate increases as the symptomatic individuals compliance increases.

### Optimal planning horizon minimizing the final epidemic size

Finally, we considered the impact of the planning horizon of non-symptomatic individuals on the final epidemic size. The planning horizon is the period over which individuals anticipate the costs and benefits of contact decisions, and the period over which they take disease prevalence to be constant. It may be thought of as the period over which individuals have confidence that the state of the epidemic will remain unchanged. We investigated the sensitivity of the attack rate to variations in the length of the planning horizon as the proportion of asymptomatic infections, and their infectiousness, change.

**Figure 5.**
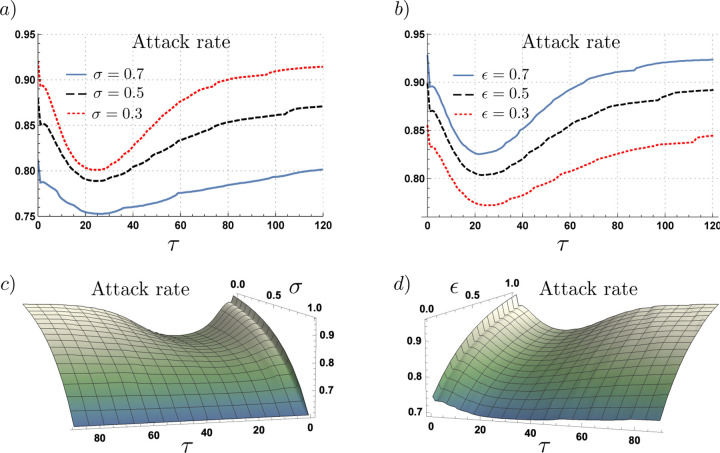
Attack rate as a function of the planning horizon for different proportion of asymptomatic cases and their relative infectiousness, under adaptive behavior model. Panels a) and b) show the non-monotonic effect of increasing the planning horizon on the attack rate, under variations of the proportion of asymptomatic cases and their relative infectiousness, respectively. Panels c) and d) shows the attack rate as a function of the planning horizon and the proportion of asymptomatic cases, and their relative infectiousness, respectively. For *τ* between 20–25 days the attack rate is minimized for all *σ* and *ε* scenarios.

We found the attack rate to be sensitive to the length of the planning horizon. Indeed, our simulations suggest there exists a planning horizon that minimizes the impact of the epidemic. Figure 5 panel a) show the attack rate as a function of the length of the planning horizon for the scenarios of 30% (*σ* = 0.3), 50% (*σ* = 0.5) and 70% (*σ* = 0.7) of asymptomatic cases, with relative infectiousness of *ε* = 0.4. Figure 5 panel b) show the attack rate as a function of the length of the planning horizon for the scenarios where asymptomatic individuals have a relative infectiousness of 70% (*ε* = 0.7), 50% (*ε* = 0.5) and 30% (*ε* = 0.3), for a proportion of asymptomatic cases of *σ* = 0.5. Our selected simulations show that the attack rate is minimized for a planning horizon between 20–25 days regardless of the proportion of asymptomatic cases and their relative infectiousness.

Figure 5 show the attack rate as a function of the planning horizon and both the proportion of asymptomatic cases where *ε* = 0.4 (panel a), and as a function of the relative infectiousness of asymptomatic individuals where *σ* = 0.5 (panel b). For the parameter values tested, the attack rate attains a minimum at 20–25 days, irrespective of the proportion of asymptomatic infections and their relative infectiousness.

The previous simulations suggest that while the projection of the benefits and costs of making contacts over a longer planning horizons is beneficial, the assumption of constant prevalence may deviate risk assessments leading to high attack rate values.

**Figure 6.**
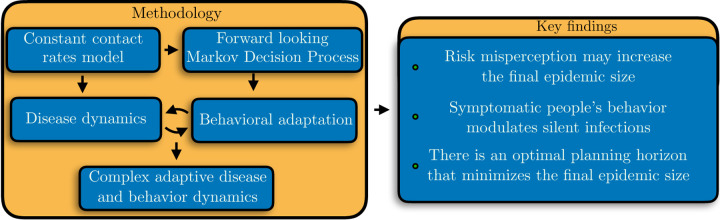
Methodology components of our adaptive behavior model and key results.

Figure 6 summarizes the methodology components of our adaptive behavior model and our key results.

## Discussion

The starting point for this analysis is the finding that adaptive behavior by non-symptomatic individuals responding to perceived infection risk alters epidemic dynamics by modifying the structure of contacts^39^. In this paper we focused on an important feature of the COVID-19 pandemic: that a large proportion of infected individuals are asymptomatic or have symptoms at a level that allows continued social interaction. Absent testing, asymptomatic individuals may both behave and be treated by others as if they are susceptible. On the other hand, absent enforcement of health authority recommendations, symptomatic individuals experiencing only mild effects may continue to engage with others.

To uncover the importance of the asymptomatic proportion of the infected population we considered the impact of behavioral responses to the risks and rewards of contact with others, assuming variable levels of compliance with health authority recommendations on the part of infected individuals. Taking the case where non-symptomatic individuals do not make any attempt to mitigate the risks to themselves or others as the base case, we considered how the inclusion of behavioral responses may be expected to alter disease dynamics. We supposed that individuals do not have perfect knowledge of either their own health class or the health class of others, and that they make decisions based on observable cues—symptoms of disease.

A study using data from New York City, New York and Austin, Texas, found that the attack rate in the first wave of the pandemic had depended on the proportion of asymptomatic infections but not on the infectiousness of asymptomatic individuals^40^. Consistent with this study, we found that while the inclusion of behavioral responses generally reduces the final epidemic size relative to the base case, the effect was highly sensitive to the proportion of the infected population that was asymptomatic. However, we also found the final epidemic size to be highly sensitive to both the infectiousness of the asymptomatic population and to the compliance with health authority recommendations of the symptomatic but socially engaged population. The higher the proportion of the infected population that is asymptomatic, and the greater the infectiousness of asymptomatic individuals, the greater the final epidemic size. Particularly, if there are asymptomatic infections, Figure 4 shows that there is a threshold determined by the proportion of asymptomatic cases and their relative infectiousness, for which the final epidemic size is larger than would occur if there were no asymptomatic infections. It also shows that the greater the rate of compliance with health authority recommendations by symptomatic individuals, the greater the likelihood that asymptomatic infections will lead to a final epidemic size larger than would occur absent asymptomatic infections.

The evidence to date on both the proportion of infections that is asymptomatic and the relative infectiousness of asymptomatics is mixed. The New York/Austin study reported that 56% of infections were estimated to be asymptomatic^40^. This result is consistent with other studies outside China^41^, but is higher than was found in studies focused on the original outbreak in Wuhan. A study of Japanese evacuees from Wuhan, for example, found the asymptomatic ratio to be 30.8%^12^.

Evidence on the relative infectiousness of symptomatic and asymptomatic individuals indicates that asymptomatic infections may well be increasing the final epidemic size. Most studies have found viral loads in symptomatic and asymptomatic individuals to be similar^42^, but even where viral loads have been found to be lower in asymptomatic individuals, a period of viral shedding has been observed^43^. Modelling exercises have shown that differences in the generation-interval distribution of asymptomatic and symptomatic transmission matter, and can significantly bias estimates of the basic reproduction number^44^. The first quantitative study of asymptomatic transmission found a total infection rate of 6.15%, with 6.30% and 4.11% for symptomatic and asymptomatic individuals respectively^45^. The implication is that the relative infectiousness of asymptomatic individuals is such that the final epidemic size is increasing in the proportion of asymptomatic infections.

Absent large scale random testing there is no way to generate precise estimates of the size of the infected and infectious asymptomatic population, and in consequence no way to generate reliable estimates of the disease reproduction number. However, by investigating changes in observable contact and associated attack rates it may be possible to infer the size and the impact of the infected asymptomatic and pre-symptomatic populations.

The framework we use to model individuals’ adaptive behavior during an epidemic focuses on the private benefits and costs of contacts. The individuals does not consider the impact that their behavior would have on others. The individual does not internalize the external costs and benefits of their behavior. The social costs of private behavior are instead reflected in health authority recommendations on, for example, social distancing measures or the use of personal protective equipment. We explore the consequences of variations in infected individuals’ willingness to comply with such recommendations. Another critical aspect on the model is the uni-dimensional and single peaked utility function. This allows us to focus on the costs and benefits of contact decisions alone, but neglects other factors that may influence individual decisions. The population in health states *S*, *E*, *A*, *R* are assumed to be homogeneous. The population in health state *I* is divided between those who choose to comply with health authority recommendations, and those who do not. They balance the costs and benefits of contact over that horizon assuming no change in prevalence. The benefits of being forward-looking in some state are constrained by the speed at which that state is changing.

While these assumptions allow us to explore the role of human behavioral responses during an epidemic, we recognize that take no account of the many other factors influencing decision-making in the current epidemic. Politicization of the epidemic is partially reflected in the parameter describing compliance with health authority recommendations, but we cannot, for example, capture the very different constraints faced by individuals in manufacturing and services, or the limited capacity to respond by those on low incomes. However, our goal is to capture the interactive evolution of human behavioral adaptation and epidemic dynamics, by using a simple but insightful mechanistic model.

## Methods

### Mathematical model

Our model focuses on infected individuals who are capable of social interaction, i.e., infected individuals who have no symptoms or mild symptoms. Since our goal is to study the impact of the behavior of infectious exposed and asymptomatic individuals on the disease dynamics, we neglect individuals with severe symptoms, since these do not interact with the rest of the population. The potential impact of nosocomial outbreaks has been analyzed in the context of SARS, pneumonia and other diseases^32^.

Our model of disease transmission is composed of susceptible (*S*), non-symptomatic and infectious exposed individuals (*E*), infected individuals with symptoms or testing positive (*I*), infectious but asymptomatic individuals (*A*), and recovered individuals (*R*). We suppose that only individuals in *I* know themselves to be infected either through observation of symptoms or through a positive test result. During the ongoing COVID-19 pandemic, it has been shown that infected individuals carry the highest viral load on or before symptom onset^33^. Due to the lack of adequate data on the specific infectiousness of exposed individuals, we assume this subpopulation to be less infectious than symptomatic infected individuals, *ρ* = 0.25. We explore the impact that changing the exposed individuals’ infectiousness produce on the evolution of the disease transmission and on the attack rate (the proportion of finally infected individuals), in the Appendix C section. We assume that on average, $\frac{1}{\kappa }$ days after infection, a proportion *σ* of exposed individuals remain asymptomatic, while the rest develop symptoms.

To capture the fact that only a fraction of the infected population will adopt pro-social precautionary behavior, we stratify the infected population into those who reduce their infectious potential by complying with health authority recommendations (*I*_*S*_), and those who do not (*I*_*C*_)^34^. We assume the fraction *l* of symptomatic individuals do not follow health authority recommendations, while the proportion 1 – *l* do it. Individuals may be non-compliant for many different reasons: they may have no reasonable alternative to interact with others, they may be compelled to continue interacting with others, they may be non-compliant for political or ideological reasons, or they may simply be careless. For our purposes all that matters is that a proportion of those known to be infected do not comply with health authority recommendations. The adoption of precautionary measures by the symptomatic population *I*_*S*_ is assumed to reduce their infectious potential by a factor *η* < 1. All other symptomatic individuals not following precautionary recommendations maintain their infectious potential. Finally, we assume a similar infectious period of $\frac{1}{\gamma }$ days for asymptomatic and symptomatic individuals.

**Figure 7.**
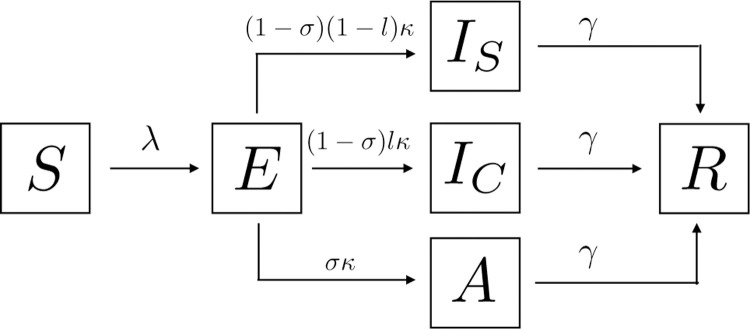
Our model for disease progression assumes susceptible (*S*), exposed (*E*), asymptomatic (*A*), symptomatic non-compliant (*I*_*C*_), symptomatic compliant (*I*_*S*_), and recovered individuals (*R*).

Our model for disease progression is sketched in Figure 7 and mathematically described by the system of equations (1)

(1) $$\begin{array}{l} \dot{S}=-\beta S\frac{\rho E+\varepsilon A+\eta I_{S}+I_{C}}{N},\\ \dot{E}=\beta S\frac{\rho E+\varepsilon A+\eta I_{S}+I_{C}}{N}-\kappa E,\\ {\dot{I}}_{S}=\left(1-\sigma \right)\left(1-l\right)\kappa E-\gamma I_{S},\\ {\dot{I}}_{C}=\left(1-\sigma \right)l\kappa E-\gamma I_{C},\\ \dot{A}=\sigma \kappa E-\gamma A,\\ \dot{R}=\gamma \left(I_{S}+I_{C}+A\right). \end{array}$$

We computed model’s (1) basic reproductive number,

(2) $$R_{0}=\beta \left(\frac{\rho }{\kappa }+\frac{\left(1-\sigma \right)\left(1-l\right)n}{\gamma }+\frac{\left(1-\sigma \right)l}{\gamma }+\frac{\sigma \varepsilon }{\gamma }\right),$$

by following the next generation matrix approach, and included the details in Appendix A.

### Disease dynamics under adaptive human behavior

Aside from the conditions that lie behind non-compliance with health authority recommendations, we assume a homogeneous population. Changes in health status are the only source of behavioral variation. Individuals’ behavior differs across health classes, but individuals with similar health status behave similarly.

Taking (1) as a baseline, we model the incidence term under adaptive behavior as

(3) $$\beta C_{t}^{S}S\frac{C_{t}^{E}\rho E+C_{t}^{A}\varepsilon A+C_{t}^{I_{S}}\eta I_{S}+C_{t}^{I_{C}}I_{C}}{C_{t}^{S}S+C_{t}^{E}E+C_{t}^{A}A+C_{t}^{I_{S}}I_{S}+C_{t}^{I_{C}}I_{C}+C_{t}^{R}R},$$

where individuals in health classes {*S*, *E*, *A*, *I*_*S*_, *I*_*C*_, *R*} select contact rates $\left\{C_{t}^{S},C_{t}^{E},C_{t}^{A},C_{t}^{I_{S}},C_{t}^{I_{C}},C_{t}^{R}\right\}$, at time *t* so as to maximize the discounted stream of net benefits–the present net value–of social interaction. Individuals make contact choices as a function of the health class-specific utility and infection risks of contacts, and this in turn influences the path of the epidemic and hence future contact risks. Observations on current disease prevalence are used to infer infection risks, and hence to project the net benefits of contact over the individual’s planning horizon. Individuals assume the population distribution among health classes and their respective current contact rates are constant over the planning horizon. Risk of infection depends on contact rates $C_{t}^{h}$ for $h\in \left\{S,E,A,I_{S},I_{C},R\right\}$, and constitutes a cost that generates a feedback between the epidemiological and economic systems. Formally, individuals in each health class maximize the expected utility of making contacts subject to the dynamics of the epidemic (7).

We determine the contact choices made by individuals at each time step, by finding the contact rate that maximize their expected utility *V*_*t*_(*h*) in each of the possible health state $h\in \left\{S,E,I_{S},I_{C},A,R\right\}$, over a given planning horizon, *τ*. At each time step, the system’s current state (population distribution among health states and their respective contact choices) is assumed to remain constant during the planning period. The expected utility *V*_*t*_(*h*) comprises the potential benefit obtained by making the optimal contact choice at each future time step during the planning horizon.

The expected utility comprises the immediate net benefits of contact (which depends only on the individual’s perceived health status), and the expected net benefits of future contacts (which depend on all possible future health states and transitions probabilities). We assume that the utility of making *C* contacts at time *t* is described by a concave single peaked utility function *u*_*t*_ = *u*(*C*_*t*_). Individuals obtain positive marginal net benefit from additional contacts up to $C_{t}^{*}$, after which additional contacts diminish the net benefits.

Following the work by Morin et. al. in^29^, we assume a utility function of the particular form $u_{t}={\left(bC_{t}^{h}-{\left(C_{t}^{h}\right)}^{2}\right)}^{v}$, where *b* is the maximum number of contacts possible, *ν* is the utility function shape parameter, and $C_{t}^{h}$ is the contact rate of a typical individual with health status *h*. Therefore, $u\left(h,C_{t}^{h}\right)$ is the utility a typical individual in health class *h* obtains by making *C* contacts at time *t*. We assume that individuals get similar per-contact utility regardless of health status, except symptomatic infected individuals who gets no utility during the infectious period. The number of daily contacts maximizing the immediate utility is given by *C*^∗^ = *b*/2.

To solve the optimization problem, we define a system of Bellman’s equations which are then numerically solved using dynamic programming methods. The detailed formulation of the decision problem is shown in Appendix B.

## Supplementary Material

Supplement

## Figures and Tables

**Table 1.** Constant contact rates and adaptive behavior model baseline parameters

Parameter	Description	Baseline value	Ref
*ν*	Utility function shape parameter	0.1	^27^
*δ*	Discount factor	0.99986	^27^
*b*	Maximum number of contacts per day	48	^27^
*β*	Likelihood of infection	0.01324	^27, 37^
*κ*	Latency rate	1/5	^4, 35^
*γ*	Recovery rate	1/9	^36^
*τ*	Planning horizon length	14	Assumed
*ρ*	Exposed ind. infectiousness	0.25	Assumed
*l*	Proportion of non-compliant ind.	1	Assumed
*η*	Compliant ind. relative infectiousness	0.4	^10^
*ε*	Asymptomatic ind. relative infectiousness	0.4	^10^
*σ*	Proportion of asymptomatic ind.	0.5	^14, 15^

## References

[1] Duong Veasna, Lambrechts Louis, Paul Richard E., Ly Sowath, Lay Rath Srey, Long Kanya C., Huy Rekol, Tarantola Arnaud, Scott Thomas W., Sakuntabhai Anavaj, and Buchy Philippe. Asymptomatic humans transmit dengue virus to mosquitoes. PNAS; Proceedings of the National Academy of Sciences, 112(47):14688–14693, 2015. 10.1073/pnas.1508114112 PMC4664300 26553981

[2] Glynn Judith R, Bower Hilary, Johnson Sembia, Houlihan Catherine F, Montesano Carla, Scott Janet T, Semple Malcolm G, Bangura Mohammed S, Kamara Alie Joshua, Kamara Osman, Asymptomatic infection and unrecognised ebola virus disease in ebola-affected households in sierra leone: a cross-sectional study using a new non-invasive assay for antibodies to ebola virus. The Lancet infectious diseases, 17(6):645–653, 2017. 28256310 10.1016/S1473-3099(17)30111-1 PMC6520246

[3] Moghadas Seyed M, Fitzpatrick Meagan C, Sah Pratha, Pandey Abhishek, Shoukat Affan, Singer Burton H, and Galvani Alison P. The implications of silent transmission for the control of covid-19 outbreaks. Proceedings of the National Academy of Sciences, 117(30):17513–17515, 2020. 10.1073/pnas.2008373117 PMC7395516 32632012

[4] Furukawa Nathan W, Brooks John T, and Sobel Jeremy. Evidence supporting transmission of severe acute respiratory syndrome coronavirus 2 while presymptomatic or asymptomatic. Emerging infectious diseases, 26(7), 2020. 10.3201/eid2607.201595 PMC7323549 32364890

[5] Gao Zhiru, Xu Yinghui, Sun Chao, Wang Xu, Guo Ye, Qiu Shi, and Ma Kewei. A systematic review of asymptomatic infections with covid-19. Journal of Microbiology, Immunology and Infection, 2020. 10.1016/j.jmii.2020.05.001 PMC7227597 32425996

[6] Li Ruiyun, Pei Sen, Chen Bin, Song Yimeng, Zhang Tao, Yang Wan, and Shaman Jeffrey. Substantial undocumented infection facilitates the rapid dissemination of novel coronavirus (sars-cov-2). Science, 368(6490):489–493, 2020. 32179701 10.1126/science.abb3221 PMC7164387

[7] Emery Jon C, Russell Timothy W, Liu Yang, Hellewell Joel, Pearson Carl AB, Knight Gwenan M, Eggo Rosalind M, Kucharski Adam J, Funk Sebastian, Flasche Stefan, The contribution of asymptomatic sars-cov-2 infections to transmission on the diamond princess cruise ship. Elife, 9:e58699, 2020. 32831176 10.7554/eLife.58699 PMC7527238

[8] Ferretti Luca, Wymant Chris, Kendall Michelle, Zhao Lele, Nurtay Anel, Abeler-Dörner Lucie, Parker Michael, Bonsall David, and Fraser Christophe. Quantifying sars-cov-2 transmission suggests epidemic control with digital contact tracing. Science, 368(6491), 2020. 10.1126/science.abb6936 PMC7164555 32234805

[9] Laxminarayan Ramanan, Wahl Brian, Dudala Shankar Reddy, Gopal K, Mohan Chandra, Neelima S, Reddy KS Jawahar, Radhakrishnan J, and Lewnard Joseph A. Epidemiology and transmission dynamics of covid-19 in two indian states. Science, 2020. 10.1126/science.abd7672 PMC7857399 33154136

[10] Oliveira Juliane F, Jorge Daniel CP, Veiga Rafael V, Rodrigues Moreno S, Torquato Matheus F, da Silva Nivea B, Fiaccone Rosemeire L, Cardim Luciana L, Pereira Felipe AC, de Castro Caio P, Mathematical modeling of covid-19 in 14.8 million individuals in bahia, brazil. Nature communications, 12(1):1–13, 2021. 10.1038/s41467-020-19798-3 PMC7803757 33436608

[11] Nogrady Bianca. What the data say about asymptomatic covid infections. Nature, 2020. 10.1038/d41586-020-03141-3 33214725

[12] Nishiura Hiroshi, Kobayashi Tetsuro, Miyama Takeshi, Suzuki Ayako, Jung Sung-mok, Hayashi Katsuma, Kinoshita Ryo, Yang Yichi, Yuan Baoyin, Akhmetzhanov Andrei R, Estimation of the asymptomatic ratio of novel coronavirus infections (covid-19). International journal of infectious diseases, 94:154, 2020. 32179137 10.1016/j.ijid.2020.03.020 PMC7270890

[13] Woloshin Steven, Patel Neeraj, and Aaron S Kesselheim. False negative tests for sars-cov-2 infection—challenges and implications. New England Journal of Medicine, 2020. 10.1056/NEJMp2015897 32502334

[14] Byambasuren Oyungerel, Cardona Magnolia, Bell Katy, Clark Justin, McLaws Mary-Louise, and Glasziou Paul. Estimating the extent of true asymptomatic covid-19 and its potential for community transmission: systematic review and meta-analysis. Available at SSRN 3586675, 2020. 10.3138/jammi-2020-0030 PMC9602871 36340059

[15] Heneghan Carl, Brassey Jon, and Jefferson Tom. Covid-19: What proportion are asymptomatic. Center for Evidence-Based Medicine, University of Oxford. https://www.cebm.net/covid-19/covid-19-what-proportion-are-asymptomatic, 2020.

[16] Meyerowitz Eric A, Richterman Aaron, Bogoch Isaac I, Low Nicola, and Cevik Muge. Towards an accurate and systematic characterisation of persistently asymptomatic infection with sars-cov-2. The Lancet Infectious Diseases, 2020. 10.1016/S1473-3099(20)30837-9 PMC7834404 33301725

[17] Mizumoto Kenji, Kagaya Katsushi, Zarebski Alexander, and Chowell Gerardo. Estimating the asymptomatic proportion of coronavirus disease 2019 (covid-19) cases on board the diamond princess cruise ship, yokohama, japan, 2020. Eurosurveillance, 25(10):2000180, 2020. 10.2807/1560-7917.ES.2020.25.10.2000180 PMC7078829 32183930

[18] Evoy David Mc, McAloon Conor G, Collins Aine B, Hunt Kevin, Butler Francis, Byrne Andrew W, Casey Miriam, Barber Ann, Griffin John M, Lane Elizabeth A, Wall Patrick, and More Simon J. The relative infectiousness of asymptomatic sars-cov-2 infected persons compared with symptomatic individuals: A rapid scoping review. medRxiv, 2020. 10.1136/bmjopen-2020-042354 PMC8098293 33947725

[19] Zhang Hong-Jun, Su Ying-Ying, Xu Shi-Lin, Chen Guo-Qing, Li Chang-Cheng, Jiang Ren-Jie, Liu Rong-Hai, Ge Sheng-Xiang, Zhang Jun, Xia Ning-Shao, Asymptomatic and symptomatic sars-cov-2 infections in close contacts of covid-19 patients: a seroepidemiological study. Clinical Infectious Diseases, 2020. 10.1093/cid/ciaa771 PMC7337633 32544949

[20] Oran Daniel P and Topol Eric J. Prevalence of asymptomatic sars-cov-2 infection: A narrative review. Annals of Internal Medicine, 2020. 10.7326/M20-3012 PMC7281624 32491919

[21] Schaller Mark. The behavioural immune system and the psychology of human sociality. Philosophical Transactions of the Royal Society B: Biological Sciences, 366(1583):3418–3426, 2011. 10.1098/rstb.2011.0029 PMC3189350 22042918

[22] Verelst Frederik, Willem Lander, and Beutels Philippe. Behavioural change models for infectious disease transmission: a systematic review (2010–2015). Journal of The Royal Society Interface, 13(125):20160820, 2016. 10.1098/rsif.2016.0820 PMC5221530 28003528

[23] Funk Sebastian, Salathé Marcel, and Jansen Vincent A. A. Modelling the influence of human behaviour on the spread of infectious diseases: a review. Journal of The Royal Society Interface, 7(50):1247–1256, 5 2010. 10.1098/rsif.2010.0142 PMC2894894 20504800

[24] Chen Jiangzhuo, Lewis Bryan, Marathe Achla, Marathe Madhav, Swarup Samarth, and Vullikanti Anil KS. Individual and collective behavior in public health epidemiology. In Handbook of statistics, volume 36, pages 329–365. Elsevier, 2017.

[25] Jackson Matthew O, Rogers Brian W, and Zenou Yves. The economic consequences of social-network structure. Journal of Economic Literature, 55(1):49–95, 2017.

[26] Granovetter Mark. The impact of social structure on economic outcomes. Journal of economic perspectives, 19(1):33–50, 2005.

[27] Fenichel Eli P, Castillo-Chavez Carlos, Ceddia M Graziano, Chowell Gerardo, Parra Paula A Gonzalez, Hickling Graham J, Holloway Garth, Horan Richard, Morin Benjamin, Perrings Charles, Adaptive human behavior in epidemiological models. Proceedings of the National Academy of Sciences, 108(15):6306–6311, 2011. 10.1073/pnas.1011250108 PMC3076845 21444809

[28] Perrings Charles, Castillo-Chavez Carlos, Chowell Gerardo, Daszak Peter, Fenichel Eli P, Finnoff David, Horan Richard D, Kilpatrick A Marm, Kinzig Ann P, Kuminoff Nicolai V, Merging economics and epidemiology to improve the prediction and management of infectious disease. EcoHealth, 11(4):464–475, 2014. 25233829 10.1007/s10393-014-0963-6 PMC4366543

[29] Morin Benjamin R, Fenichel Eli P, and Castillo-Chavez Carlos. Sir dynamics with economically driven contact rates. Natural resource modeling, 26(4):505–525, 2013. 25152563 10.1111/nrm.12011 PMC4139939

[30] Rader Benjamin, White Laura F, Burns Michael R, Chen Jack, Brilliant Joe, Cohen Jon, Shaman Jeffrey, Brilliant Larry, Hawkins Jared B, Scarpino Samuel V, Mask wearing and control of sars-cov-2 transmission in the united states. medRxiv, 2020. 10.1016/S2589-7500(20)30293-4 PMC7817421 33483277

[31] Feng Shuo, Shen Chen, Xia Nan, Song Wei, Fan Mengzhen, and Benjamin J Cowling. Rational use of face masks in the covid-19 pandemic. The Lancet Respiratory Medicine, 8(5):434–436, 2020. 32203710 10.1016/S2213-2600(20)30134-X PMC7118603

[32] Brauer Fred. Some simple nosocomial disease transmission models. Bulletin of Mathematical Biology, 77(3):460–469, 2015. 25608612 10.1007/s11538-015-0061-0

[33] He Xi, Lau Eric H. Y., Wu Peng, Deng Xilong, Wang Jian, Hao Xinxin, Lau Yiu Chung, Wong Jessica Y., Guan Yujuan, Tan Xinghua, Mo Xiaoneng, Chen Yanqing, Liao Baolin, Chen Weilie, Hu Fengyu, Zhang Qing, Zhong Mingqiu, Wu Yanrong, Zhao Lingzhai, Zhang Fuchun, Cowling Benjamin J., Li Fang, and Leung Gabriel M. Temporal dynamics in viral shedding and transmissibility of COVID-19. Nature Medicine, 26(5):672–675, apr 2020. 10.1038/s41591-020-0869-5 32296168

[34] Acuña-Zegarra Manuel Adrian, Santana-Cibrian Mario, and Velasco-Hernandez Jorge X. Modeling behavioral change and covid-19 containment in mexico: A trade-off between lockdown and compliance. Mathematical Biosciences, page 108370, 2020. 32387384 10.1016/j.mbs.2020.108370 PMC7202859

[35] Lipsitch Marc, Cohen Ted, Cooper Ben, Robins James M, Ma Stefan, James Lyn, Gopalakrishna Gowri, Chew Suok Kai, Tan Chorh Chuan, Samore Matthew H, Transmission dynamics and control of severe acute respiratory syndrome. science, 300(5627):1966–1970, 2003. 12766207 10.1126/science.1086616 PMC2760158

[36] Wölfel Roman, Corman Victor M, Guggemos Wolfgang, Seilmaier Michael, Zange Sabine, Müller Marcel A, Niemeyer Daniela, Jones Terry C, Vollmar Patrick, Rothe Camilla, Virological assessment of hospitalized patients with covid-2019. Nature, 581(7809):465–469, 2020. 32235945 10.1038/s41586-020-2196-x

[37] Shi Qiuling, Hu Yaoyue, Peng Bin, Tang Xiao-Jun, Wang Wei, Su Kun, Luo Chao, Wu Bo, Zhang Fan, Zhang Yong, Effective control of sars-cov-2 transmission in wanzhou, china. Nature medicine, 27(1):86–93, 2021. 10.1038/s41591-020-01178-5 33257893

[38] Zhao Shi, Lin Qianyin, Ran Jinjun, Musa Salihu S, Yang Guangpu, Wang Weiming, Lou Yijun, Gao Daozhou, Yang Lin, He Daihai, Preliminary estimation of the basic reproduction number of novel coronavirus (2019-ncov) in china, from 2019 to 2020: A data-driven analysis in the early phase of the outbreak. International journal of infectious diseases, 92:214–217, 2020. 32007643 10.1016/j.ijid.2020.01.050 PMC7110798

[39] Fenichel Eli P. Economic considerations for social distancing and behavioral based policies during an epidemic. Journal of health economics, 32(2):440–451, 2013. 23419635 10.1016/j.jhealeco.2013.01.002 PMC3659402

[40] Fox Spencer J, Pasco Remy, Tec Mauricio, Du Zhanwei, Lachmann Michael, Scott James, and Meyers Lauren Ancel. The impact of asymptomatic covid-19 infections on future pandemic waves. medRxiv, 2020.

[41] Kronbichler Andreas, Kresse Daniela, Yoon Sojung, Lee Keum Hwa, Effenberger Maria, and Shin Jae Il. Asymptomatic patients as a source of covid-19 infections: A systematic review and meta-analysis. International journal of infectious diseases, 98:180–186, 2020. 32562846 10.1016/j.ijid.2020.06.052 PMC7832751

[42] Wang Yishan, Kang Hanyujie, Liu Xuefeng, and Tong Zhaohui. Asymptomatic cases with sars-cov-2 infection. Journal of Medical Virology, 2020. 10.1002/jmv.25990 PMC7267605 32383171

[43] Zhou Rui, Li Furong, Chen Fengjuan, Liu Huamin, Zheng Jiazhen, Lei Chunliang, and Wu Xianbo. Viral dynamics in asymptomatic patients with covid-19. International Journal of Infectious Diseases, 2020. 10.1016/j.ijid.2020.05.030 PMC7211726 32437933

[44] Park Sang Woo, Cornforth Daniel M, Dushoff Jonathan, and Weitz Joshua S. The time scale of asymptomatic transmission affects estimates of epidemic potential in the covid-19 outbreak. Epidemics, page 100392, 2020. 32446187 10.1016/j.epidem.2020.100392 PMC7212980

[45] Wu ZY. Asymptomatic and pre-symptomatic cases of covid-19 contribution to spreading the epidemic and need for targeted control strategies. Zhonghua liu Xing Bing xue za zhi, 41:E036–E036, 2020. 10.3760/cma.j.cn112338-20200406-00517 32274917

[46] Diekmann O., Heesterbeek J. A. P., and Metz J. A. J. On the definition and the computation of the basic reproduction ratio R0 in models for infectious diseases in heterogeneous populations. J. Math. Biol., 28(4):365–382, 1990. 2117040 10.1007/BF00178324

[47] van den Driessche P. and Watmough J. reproduction numbers and sub-threshold endemic equilibria for compartmental models of disease transmission. Math. Biosci., 180:29–48, 2002. 12387915 10.1016/s0025-5564(02)00108-6

[48] Wise Toby, Zbozinek Tomislav D, Michelini Giorgia, Hagan Cindy C, and Mobbs Dean. Changes in risk perception and self-reported protective behaviour during the first week of the covid-19 pandemic in the united states. Royal Society Open Science, 7(9):200742, 2020. 33047037 10.1098/rsos.200742 PMC7540790

