# Supplementary material for: Adaptive human behavior in epidemics: the impact of risk misperception on the spread of epidemics
Source: Res Sq. 2021 Feb 23:rs.3.rs-220733. Preprint. [Version 1] doi: 10.21203/rs.3.rs-220733/v1 (PMC7924275; doi:10.21203/rs.3.rs-220733/v1)
Supplement: Supplement [file 28ef2134edbd12ce6535f3ad.pdf]

Supplementary Material

Adaptive human behavior in epidemics: the impact of risk misperception on the spread of epidemics

Baltazar Espinoza[1], Madhav Marathe[1] Samarth Swarup[1], Mugdha Thakur[1]

1. Biocomplexity Institute and Initiative, Network Systems Science and Advanced Computing Division, University of Virginia, Virginia, USA

A Constant contacts model's basic reproductive number

We use the next generation approach [1, 2], to compute Model's (1) basic reproductive number. We consider the infectious compartments E, I and A and define

$$\mathcal{F} = \begin{pmatrix} \beta S \frac{\rho E + \varepsilon A + \eta I_S + I_C}{N} \\ 0 \\ 0 \\ 0 \end{pmatrix} \quad \text{and} \quad \mathcal{V} = \begin{pmatrix} -\kappa E \\ \gamma I_S - (1-\sigma)(1-l)\kappa E \\ \gamma I_C - (1-\sigma) l \kappa E \\ \gamma A - \sigma \kappa E \end{pmatrix},$$

where the next generation matrix is given by $-FV^{-1}|_{S=N}$ and the basic reproductive number given by its spectral radius

$$\mathcal{R}_0 = \beta \left(\frac{\rho}{\kappa} + \frac{(1-\sigma)(1-l)\eta}{\gamma} + \frac{(1-\sigma)l}{\gamma} + \frac{\sigma\varepsilon}{\gamma} \right), \tag{A.1}$$

with F and V representing the Jacobian matrices of $\mathcal{F}$ and ν, respectively. The basic reproductive number (A.1) accounts for the average secondary infections produced by exposed $\left(\beta \frac{\rho}{\kappa}\right)$, symptomatic compliant $\left(\beta \frac{(1-\sigma)(1-l)\eta}{\gamma}\right)$, symptomatic non-compliant $\left(\beta \frac{(1-\sigma)l}{\gamma}\right)$ and asymptomatic individuals $\left(\beta \frac{\sigma\varepsilon}{\gamma}\right)$. Notice that in the absence of compliant individuals ($l = 1$), asymptomatic infections ($\sigma = 0$), and infectious exposed individuals ($\rho = 0$), the basic reproductive number takes the traditional form $\mathcal{R}_0 = \frac{\beta}{\gamma}$.

During the initial stage of an epidemic, the disease initially propagates in the absence of sanitary recommendations on a population almost completely susceptible. In the absence of human adaptive response, the potential of an epidemic to propagate among the population is captured by its basic reproductive number. We use expression (A.1) to explore the trade-off between the proportion of initially asymptomatic infections and the proportion of symptomatic individuals that makes an epidemic mathematically sustainable ($\mathcal{R}_0 > 1$), in the absence of behavioral response.

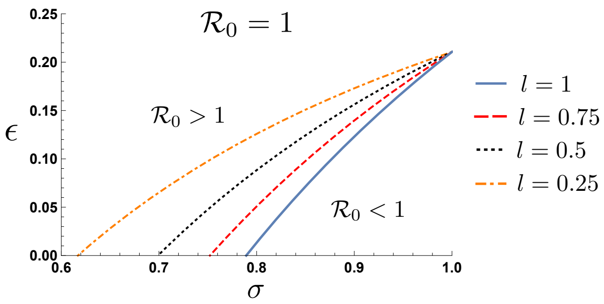

Figure A.1: The trade-off between the proportion of asymptomatic and their relative infectiousness producing $\mathcal{R}_0 = 1$, for scenarios where of 25%, 50%, 75% and 100% of the symptomatic individuals are non-compliant ($l = 0.25, 0.5, 0.75$ and $l = 1$, respectively). A COVID-19 like epidemic, initially propagating in the absence of sanitary recommendations ($l = 1$ and $\mathcal{R}_0 = 2.4$), is mathematically sustainable whenever the proportion of asymptomatic cases is less than 80%, regardless of their relative infectiousness. Parameter values: $\gamma = \frac{1}{9}, \kappa = \frac{1}{5}, \beta = 0.01324, C_t = 48, \rho = 0.25$ and $\eta = 0.4$.

Figure A.1 shows the level curves $\mathcal{R}_0 = 1$ as a function of the proportion of asymptomatic individuals (σ) and their relative infectiousness (ϵ), for scenarios where where of 25%, 50%, 75% and 100% of the symptomatic individuals are non-compliant (l). Our simulations show that a COVID-19 like epidemic, initially propagating in the absence of sanitary recommendations ($l = 1$ and $\mathcal{R}_0 = 2.4$), is mathematically sustainable whenever the proportion of asymptomatic cases is less than 80%, regardless of their relative infectiousness. In other words, at the early stage of the epidemic, a force of infection driven solely by symptomatic cases is enough to propagate the disease among the population.

B A model of disease risk mitigation

Non-symptomatic individuals behavior

In the absence of symptoms, we assume exposed and asymptomatic individuals are not aware of their infectious status, perceiving themselves to be susceptible. In consequence, we suppose that non-symptomatic individuals in all three health classes –susceptible, exposed and asymptomatic– choose their contact rates in the same way. All non-symptomatic individuals choose the contact rate that maximizes expected utility over the planning horizon $[t, t+\tau]$. This is done by weighing current and the expected future benefits of contact against the risk of infection. Expected benefits are conditioned on the probability of future infection, and potential recovery. We model the optimization problem as a dynamic programming problem, the solution to which generates the privately optimal contact rate, [4, 5, 3].

Formally, the dynamic programming problem by which susceptible individuals assess the daily optimal contact rate is given by the Bellman equation

$$V_t(S) = \max_{C_t^S}\{u(S, C_t^S) + \delta[(1 - P^I)V_{t+1}(S) + P^I(V_{t+1}(E))]\} \tag{B.1}$$

where $V_t(S)$ is the expected utility of susceptible individuals at time t, $V_{t+1}(S)$ ($V_{t+1}(E)$) is the expected utility being susceptible (exposed) at time $t+1$, and

$$P^I = \exp\left(\beta S \frac{\rho E + \varepsilon A + \eta I_S + I_C}{N}\right)$$

is the probability of being infected at time t.

The maximization problem in equation (B.1) accounts for the individual's immediate utility ($u(S, C_t^S)$) plus the expected future utility discounted at rate δ. The susceptible individual's expected future utility comprises the expected utility of remaining susceptible with probability $1 - P^I$ and, the expected utility of being infected (progressing to the E compartment) with probability P^I.

Notice that in order to solve equation (B.1), the expected utility of exposed individuals is required, which is given by equation (B.2)

$$V_t(E) = u(E, C_t^S) + \delta[(1 - P^E)V_{t+1}(E) \ + \ P^E(\sigma V_{t+1}(A) + (1-\sigma)[(1-l)V_{t+1}(I_S) + lV_{t+1}(I_C)])], \tag{B.2}$$

where $P^E = e^{-\kappa}$ stands for the probability of moving from the E health class to either A, I_S or I_C health classes, with probabilities defined by our constant contact rates model. Similar to equation (B.1), $V_t(E)$ sums the immediate utility of currently being exposed ($u(E, C_t^S)$) and the discounted expected future utility of progressing to possible future health states. The future expected utility while exposed comprises the expected utility of remaining in the exposed compartment with probability $(1-P^E)$ or progressing out of the exposed compartment with probability P^E. The future expected utility for exposed individuals progressing to a different health class comprises the future expected utilities of being asymptomatic, infected compliant or infected non-compliant, with probabilities $P^E\sigma, P^E(1-\sigma)(1-l)$ and $P^E(1-\sigma)l$, respectively.

Finally, the expected utility of asymptomatic ($V_t(A)$), infected compliant ($V_t(I_S)$) and infected non-compliant ($V_t(I_C)$), comprise the immediate utility and the discounted future expected utility when recovered ($V_t(R)$). The Bellman equations for individuals in these health states are, respectively:

$$V_t(A) = U(A, C_t^S) + \delta[P^R V_{t+1}(A) + (1 - P^R)V_{t+1}(R)], \tag{B.3}$$

$$V_t(I_S) = U(I_S, C_t^{I_S}) + \delta[P^R V_{t+1}(I_S) + (1 - P^R)V_{t+1}(R)], \tag{B.4}$$

$$V_t(I_C) = U(I_C, C_t^{I_C}) + \delta[P^R V_{t+1}(I_C) + (1 - P^R)V_{t+1}(R)], \tag{B.5}$$

where $P^R = e^{-\gamma}$ is the probability of recovery.

Notice that the assumption that non-symptomatic individuals are unaware of their health status implies that current utility ($U(h, C_t^S)$, $h \in \{E, A\}$), is maximized by choosing a contact rate that corresponds to the susceptible health state. That is, the contact rates used in the terms $u(E, C_t^S)$ and $u(A, C_t^S)$ in equations (B.2) and (B.3), respectively, are driven by individuals' own health status perception.

Symptomatic infected individuals

We suppose that symptomatic infected individuals divide into two sub-classes: a fraction $1 - l$ of symptomatic individuals comply with health authority recommendations for the mitigation of population level disease risk (I_S), while the rest of symptomatic individuals do not comply with those recommendations (I_C). We suppose that all individuals in I_S and I_C, develop symptoms and are aware that they are infected and infectious. The solution to the Bellman equation for symptomatic infected individuals generates the privately optimal contact rate for individuals in that health class. However, we also suppose that individuals in I_S are willing to reduce their contact rate below the privately optimal level in compliance with health authority recommendations [6].

More particularly, we suppose that compliant infected individuals are willing to accept a reduction in the utility they gain from contacts, so long as utility does not fall below the minimum acceptable level, u_c. In this respect the approach differs from the framework proposed in [4, 3, 5].

Note that expected utility in (B.4) and (B.5) depends on the average recovery period. We therefore derive the following explicit expression for non-compliant I_C individuals' expected utility

$$V_t(I_C) = u(I_C, C^{I_C*}) \sum_{j=1}^{\tau} \delta^j (1 - P^R)^j + u(R, C^{R*}) \sum_{j=1}^{\tau} \delta^j \left(1 - (1 - P^R)^j\right), \tag{B.6}$$

where $C_t^{I_C*} \leq C_t^*$. The first term of (B.6) corresponds to the expected utility obtained while infected, and the second term corresponds to the expected utility obtained while recovered, during the planning horizon.

By contrast, compliant individuals reduce their contact rate subject to a level consistent with securing a minimal utility, so solving the problem

$$V_t(I_S) = \min_{C_t^{I_S}} \{u(I_S, C_t^{I_S}) \sum_{j=1}^{\tau} \left(\delta(1 - P^R)\right)^j, \ \text{ subject to } \ \frac{V_t(I_S)}{\tau} > u_C. \tag{B.7}$$

Notice that equation (B.7) comprises only the infectious period, since an infected individual is assumed to stop following this behavioral regime when recovered.

Recovered individuals

We assume there is no incentive for recovered individuals to behave strategically. Therefore, we let recovered individuals make the daily number of contacts that maximizes the net benefits of contact. The recovered individuals Bellman equation is given by

$$V_t(R) = u(R, C_t^{R*}) + \delta V_{t+1}(R). \tag{B.8}$$

Optimal behavioral decisions via Bellman's equations and dynamic programming

In this section we illustrate the dynamic programming method we use to solve equations (B.1)-(B.8) via backward induction over the planning horizon $[t, t+\tau]$. In order to obtain the optimal contact rate at current time t, we make use of the *Bellman's principle of optimality.* The dynamic optimization problem over the whole planning horizon is split into a sequence of subproblems over sequencial periods $[t, t+1]$, $[t+1, t+2]$,$\cdots$,$[t+\tau-1, t+\tau]$.

To solve the dynamic programming problem backwards on time we need boundary conditions. Notice that the following relations holds regardless of the individual's health status h,

- $V_{t+\tau+1}(h) = 0$, since it exceeds the boundary of the planning horizon $[t, t+\tau]$,
- $V_{t+\tau}(h) = u_{t+\tau}(h)$, since it is the last period of the planning horizon.

Now, let's analyze backwards induction method to solve susceptible individuals' Bellman's equation. To find the optimal contact rate at current time, we assume a constant projection of the system's current state.

- **Susceptible individual's optimal contact choice at time $t+\tau$**
  Since this is the last period of the planning horizon, individuals do not assess future risk or benefits beyond the current day. Therefore the optimal decision at $t+\tau$ is the contact rate that maximizes individuals' immediate utility,
$$V_{t+\tau}(S) = u(S, C^*), \quad \text{and} \quad V_{t+\tau}(E) = u(E, C^*)$$
  where $C^* = b/2$.

- **Susceptible individual's optimal contact choice at time $t+\tau-1$**
  To find the optimal contact rate at this period we should make use of the previously found optimal choice at time $t+\tau$. The optimization problem becomes
$$\begin{aligned} V_{t+\tau-1}(S) &= \max_{C^S_{t+\tau-1}} \{u(S, C^S_{t+\tau-1}) + \delta[(1-P^I)V_{t+\tau}(S) + P^I(V_{t+\tau}(E))]\}, \\ &= \max_{C^S_{t+\tau-1}} \{u(S, C^S_{t+\tau-1}) + \delta[(1-P^I)u(S,C^*) + P^I(u(E,C^*))]\}, \end{aligned}$$
  finally
$$V_{t+\tau-1}(S) = \max_{C^S_{t+\tau-1}} \{u(S, C^S_{t+\tau-1}) + \delta[(1-P^I)u(S,C^*) + P^I(u(E,C^*))]\} \tag{B.9}$$
  where P^I (the probability of being infected) is known from the system's state at time t
$$P^I = 1 - \exp\left(-\beta S \frac{\rho E + \varepsilon A + \eta I_S + I_C}{N}\right).$$
  The optimal contact rate for the period $t+\tau-1$, can be obtained from equation (B.9) by evaluating all the possible contact rates.

- **Susceptible individual's optimal contact choice at time $t+\tau-2$**
  To find the optimal contact rate at this period we should make use of the optimal choices at $t+\tau$ and $t+\tau-1$. The problem becomes
$$V_{t+\tau-2}(S) = \max_{C^S_{t+\tau-2}} \{u(S, C^S_{t+\tau-2}) + \delta[(1-P^I)V_{t+\tau-1}(S) + P^I(V_{t+\tau-1}(E))]\}, \tag{B.10}$$
  notice that we require $V_{t+\tau-1}(E)$ to solve equation (B.10). Here we make use of the Bellman's equation for exposed individuals, at the period $t+\tau-1$,
$$\begin{aligned} V_{t+\tau-1}(E) =& u(E, C^S_{t+\tau-1}) + \delta[(1-P^E)V_{t+\tau}(E) \\ &+ P^E(\sigma V_{t+\tau}(A) + (1-\sigma)[(1-l)V_{t+\tau}(I_S) + lV_{t+\tau}(I_C)])], \\ =& u(E, C^S_{t+\tau-1}) + \delta[(1-P^E)u(E,C^*) \\ &+ P^E(\sigma u(A,C^*) + (1-\sigma)[(1-l)u(I_S,C^*) + lu(I_C,C^*)])], \end{aligned}$$
  that is
$$\begin{aligned} V_{t+\tau-1}(E) =& u(E, C^S_{t+\tau-1}) + \delta[(1-P^E)u(E,C^*) \\ &+ P^E(\sigma u(A,C^*) + (1-\sigma)[(1-l)u(I_S,C^*) + lu(I_C,C^*)])]. \end{aligned} \tag{B.11}$$
  Notice that we use $u(E, C^E_{t+\tau-1}) = u(E, C^S_{t+\tau-1})$ since we assume exposed individuals are unaware of their health status and chose the contact rate that corresponds to the susceptible health state. Therefore, $V_{t+\tau-1}(E)$ is known, and equation (B.10) can also be solved by optimizing over all the possible contact rates.

By continuing the backward induction it is possible to solve for the optimal contact rate at each time during the planning horizon, and particularly for the current time t. Since in our epidemic model we only care about

the optimal contact rate for the period $t+1$, we use C^S_{t+1} as the contact rate chosen by susceptible and non-symptomatic individuals to run the epidemic model one period ahead.

Table B.1: Recursive dependence of the optimization problem

Health class	$t+\tau-3$	$t+\tau-2$	$t+\tau-1$	$t+\tau$
S	S, E, I_S, I_C, A, R	S, E, I_S, I_C, A	S, E	S
E	S, E, I_S, I_C, A, R	S, E, I_S, I_C, A, R	S, E, I_S, I_C, A	E
I_S	I_S, R	I_S, R	I_S, R	I_S
I_C	I_C, R	I_C, R	I_C, R	I_C
A	A, R	A, R	A, R	A
R	R	R	R	R

C Sensitivity analysis

Summary

Table C.1: Sensitivity summary

Parameter	Description	Range
ρ	Exposed ind. infectiousness	[0, 0.5]
b	Maximum number of contacts per day	[24, 48]
ν	Utility function shape parameter	[0.05, 0.2]
δ	Discount factor	[0.99939, 0.99986]

- Increments in the exposed individuals infectiousness ρ
  - Increases the peak size and the attack rate.
  - Reduces the peak time.
  - Produce stronger adaptive response: increases contacts reduction during the peak time.
  - Reduces the asymptomatic infectiousness ϵ for which the attack rate overcome the attack rate in the absence of asympto
- Maximum number of daily contacts b
  - We found the adaptive behavior model to be low sensitive to changes in b.
- Increments in the utility function shape parameter ν
  - Increase immediate utility and the marginal benefit of increasing contacts.
  - Delay the adaptive response and, in consequence the peak time and the attack rate.
  - Produce a weaker adaptive response: lower reduction of contacts.
- Increments in the annual discount rate δ
  - We found the adaptive behavior model to be low sensitive to changes in δ.

Exposed individuals infectiousness ρ

Since the infectiousness of infectious pre-symptomatic individuals is unknown, for our simulations we assumed the exposed sub-population have a reduced infectious rate relative to symptomatic individuals, $\rho = 0.25$. In this section we aim to study the impact of variations in the relative infectiousness of exposed individuals on the evolution of disease transmission and the final epidemic size.

Selected simulations in Figure C.1 show the impact of the relative infectiousness of exposed individuals on the evolution of the disease dynamics for the constant contacts model (dashed lines) and the adaptive behavior model (solid lines). We explored the scenarios where exposed individuals are 0%, 25% and 50% as infectious as symptomatic individuals, for the set of parameters in Table ?? with $\sigma = 0.3$ and $\epsilon = 0.6$.

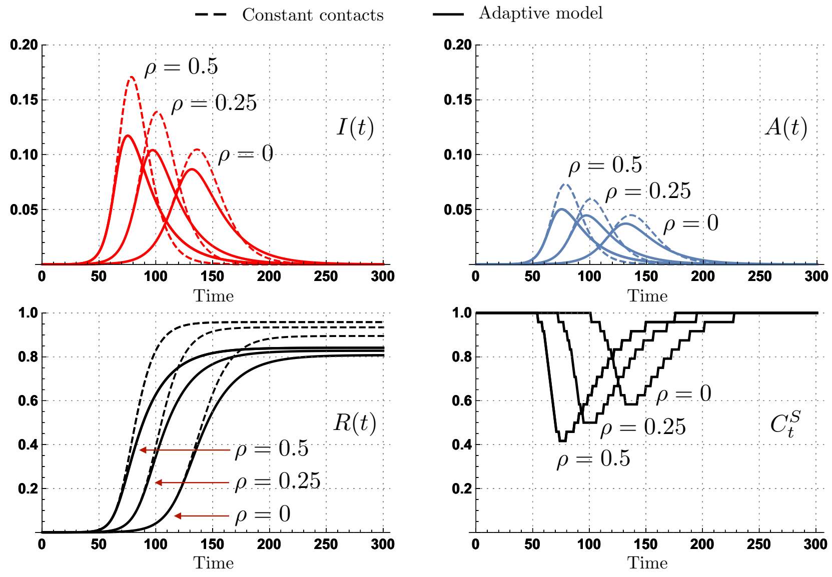

Figure C.1: Disease dynamics under constant contact rates (dashed lines) and under adaptive behavior (solid lines). Exposed individuals' relative infectiousness of $\rho = 0, 0.25$ and 0.5, for the parameters set in Table (1) with $\sigma = 0.3$ and $\epsilon = 0.6$. Increments on the exposed individuals' relative infectiousness increases the peak size and decreases the peak time. Moreover, the final epidemic size is increases and the behavioral response

Greater exposed individuals' relative infectiousness increases the peak size and the final epidemic size, while decreasing the peak time. In these scenarios, the adaptive response is triggered earlier but at the same prevalence level. Moreover, higher ρ values lead to greater reduction of contacts.

In Figure C.2 we explore the impact of changes in the relative infectiousness of exposed individuals on the attack rate, for the cases where $\rho = 0, 0.25$ and 0.5. We focus on the (σ, ϵ) scenarios producing an attack rate greater than the one corresponding to the baseline scenario, no asymptomatic infections ($\sigma = 0$).

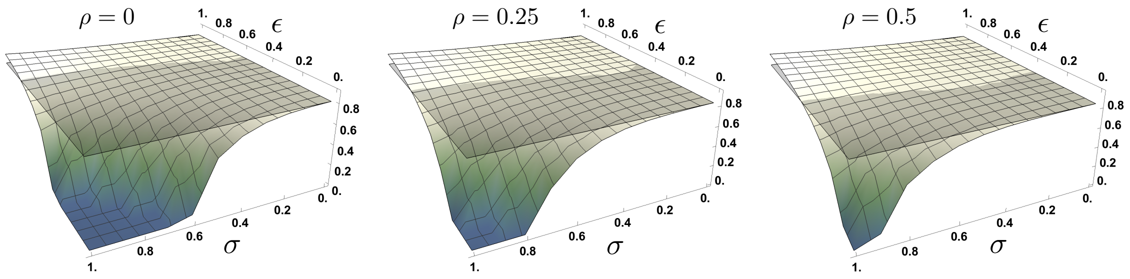

Figure C.2: Attack rate as a function of the proportion of asymptomatic infections (σ) and their relative infectiousness (ϵ), for exposed individuals infectiousness of $\rho = 0, 0.25$ and 0.5. The greater the exposed individuals' infectiousness the lower the asymptomatic individuals' infectiousness required to have an attack rate over the baseline scenario.

Figure C.2 shows that the greater the infectiousness of exposed individuals, the greater the attack rate for all (σ, ϵ) scenarios. Moreover, greater ρ values reduce the asymptomatic individuals' infectiousness (ϵ) required to produce an attack rate above the baseline scenario (gray plane). In other words, the impact of risk misperception increases as the infectiousness of non-symptomatic individuals increases. In addition, high infectiousness of exposed individuals increase the (σ, ϵ) scenarios for which an outbreak is produced.

The utility function $u(C) = \left(bC - C^2\right)^{\nu}$

Due to the absence of appropriate data to calibrate our behavior model, specifically the parameters used in the utility function, in this section we test the sensitivity of the behavioral response and disease dynamics to

changes in the utility parameters. We found the disease dynamics obtained with the behavior model to be low sensitive to the assumed amount of available contacts per day, (b). For an assumed b value we adjust the per-capita likelihood of infection β, so that the basic reproductive number of the behavior model (C.1), matches the targeted basic reproductive value of 2.4,

$$\mathcal{R}_0(C) = C^*\beta\left(\frac{\rho}{\kappa} + \frac{(1-\sigma)(1-l)\eta}{\gamma} + \frac{(1-\sigma)l}{\gamma} + \frac{\sigma\varepsilon}{\gamma}\right), \tag{C.1}$$

where C^* corresponds to the optimal contact rate in the absence of disease.

Figure C.3 shows the disease dynamics for the constant contact rate (dashed lines) and for the behavior model (solid lines), for the scenarios where the maximum daily contact rate of 48 and a per-contact likelihood of infection $\beta = 0.01324$ (panel a), maximum daily contact rate of 24 and a per-contact likelihood of infection $\beta = 0.02649$ (panel b). We found the disease dynamics and the behavioral response produced in both scenarios to be equivalent. Both scenarios produce an attack rate of about 80% and the behavioral response reduce the amount of contacts to 50% during the peak of the epidemic. That is, the higher the amount of contacts assumed, the higher the resolution of the behavioral change over time obtained.

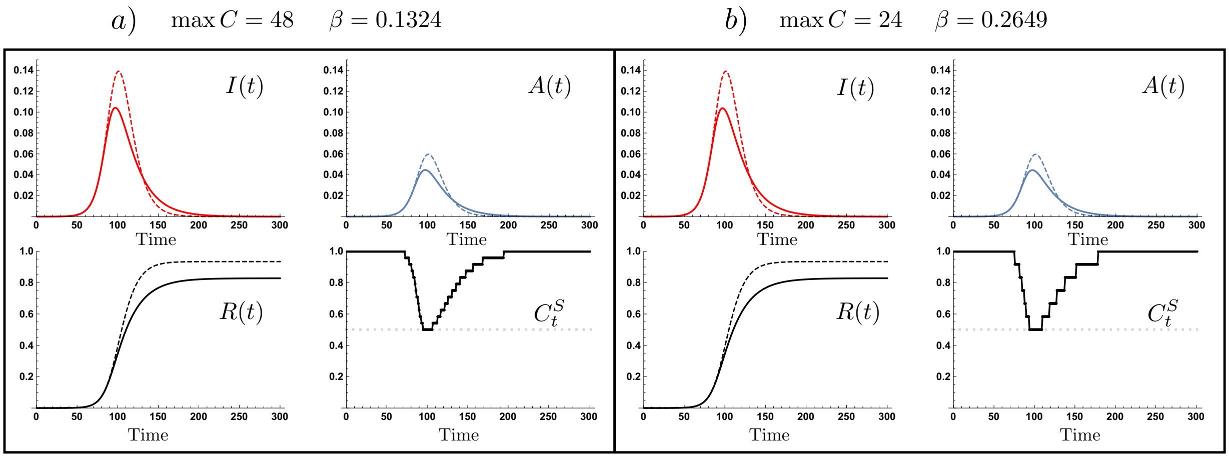

Figure C.3: Disease dynamics under adaptive behavior for different contact rates: in panel a) the maximum contact rate of $b = 48$ leads to $\beta = 0.01324$, in panel b) the maximum contact rate $b = 24$ leads to $\beta = 0.02649$. The normalized contact rate curves in both scenarios $\left(C_t/C^*\right)$ is taken down to a 50% reduction of contacts during the peak of the outbreak. In both scenarios, parameter sets produce a basic reproductive value is $\mathcal{R}_0 = 2.4$. In both scenarios the adjusted basic reproductive values is $\mathcal{R}_0 = 2.4$. For parameters in Table (1) with $\sigma = 0.3$ and $\epsilon = 0.6$

Utility function shape parameter ν

In the proposed behavior model, adaptive response is triggered by assessing the benefits of making contacts while being exposed to the risk of infection. Due to the lack of appropriate data to calibrate the utility function, for our numerical experiments we arbitrarily chose $\nu = 0.1$. Here, we explore the impact that variations in the utility function shape parameter (ν) produce on the disease dynamics.

Figure C.4 panel a) shows the immediate utility obtained by individuals making C contacts per day for ν values of 0.05, 0.1 and 0.2, and where the maximum number of contacts per day is $b = 48$. Lower ν values, makes contacts to be less valuable by producing a lower utility ($u(C, \nu_1) > u(C, \nu_2)$ for $\nu_1 > \nu_2$). We found the behavior model to be highly sensitive to this parameter since the risk-benefit trade-off is directly impacted by the utility obtained by making contacts. Specifically, reducing the amount of utility obtained results in an earlier start of behavioral response. Moreover, Figure C.4 panel b) shows that low ν values reduces the marginal benefit of increasing contacts. Producing an stronger adaptive response since for lower ν values it is "cheaper" to modify contact rates, as compared to bigger ν values.

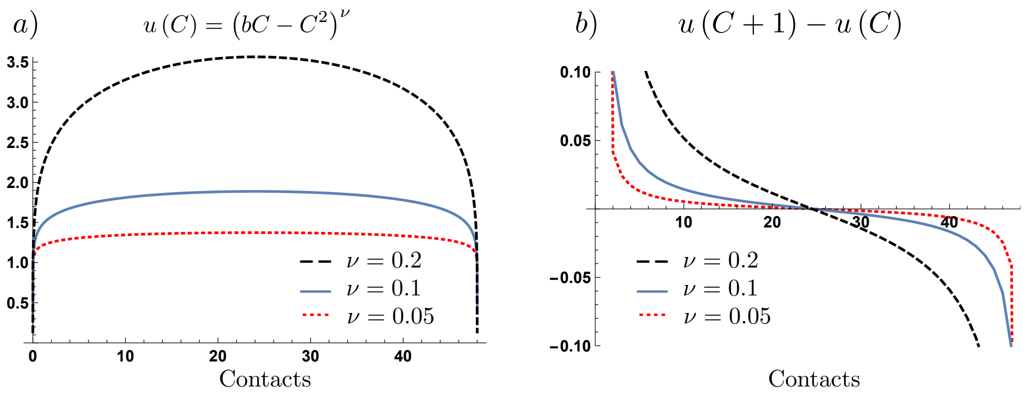

Figure C.4: Figure shows the immediate utility obtained by individuals making C contacts (panel a) and the marginal benefit of increasing contacts (panel b), for ν values of 0.05, 0.1 and 0.2, and maximum daily contacts of $b = 48$. The immediate utility obtained by making C contacts and the marginal benefit of increasing contacts decrease as the ν value decreases.

Figure C.5 show the disease dynamics and adaptive response obtained for the constant contact rates model (dashed lines), and for the adaptive behavior model, using the utility shape parameter values $\nu = 0.05, 0.1$ and 0.2, (solid, dotted, and dot-dashed lines respectively).

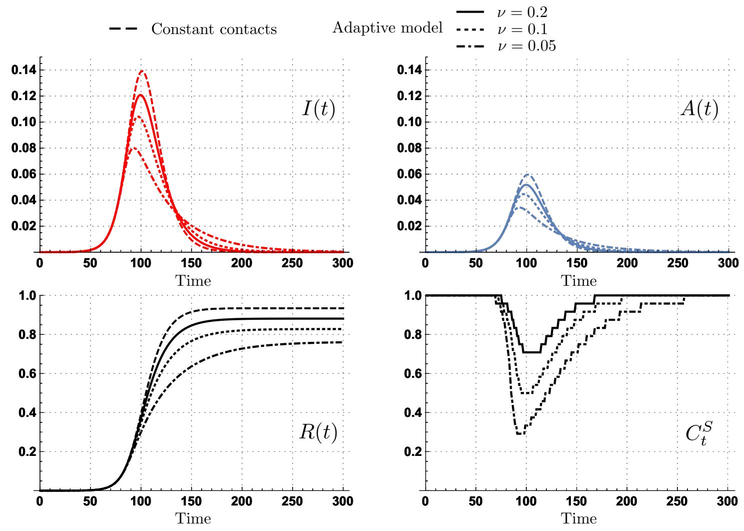

Figure C.5: Disease dynamics under constant contact rates (dashed lines) and under adaptive behavior, for utility shape parameter $\nu = 0.05, 0.1$ and 0.2 (solid, dotted and dot-dashed lines respectively), for parameters in Table (1) with $\sigma = 0.3$ and $\epsilon = 0.6$. Reductions in the utility shape function parameter (ν), produces an earlier start of behavioral adaptation as well as a greater reduction in the contacts during the outbreak.

Reductions on the utility shape function parameter (ν), highly impact the disease dynamics and the adaptive response produced. Decreasing the ν value reduces the amount of utility obtained per contact as shown in Figure C.4 panel a). In consequence starting the adaptive response earlier and producing a greater contacts curtail. This impacts the disease dynamics in several ways, the peak size and the final epidemic size substantially decrease. However, the epidemic period is increased and the peak time is reduced.

References

[1] O. Diekmann, J. A. P. Heesterbeek, and J. A. J. Metz. On the definition and the computation of the basic reproduction ratio R_0 in models for infectious diseases in heterogeneous populations. *J. Math. Biol.*, 28(4):365–382, 1990.

[2] P. van den Driessche and J. Watmough. reproduction numbers and sub-threshold endemic equilibria for compartmental models of disease transmission. *Math. Biosci.*, 180:29–48, 2002.

[3] Benjamin R Morin, Eli P Fenichel, and Carlos Castillo-Chavez. Sir dynamics with economically driven contact rates. *Natural resource modeling*, 26(4):505–525, 2013.

[4] Eli P Fenichel, Carlos Castillo-Chavez, M Graziano Ceddia, Gerardo Chowell, Paula A Gonzalez Parra, Graham J Hickling, Garth Holloway, Richard Horan, Benjamin Morin, Charles Perrings, et al. Adaptive human behavior in epidemiological models. *Proceedings of the National Academy of Sciences*, 108(15):6306–6311, 2011.

[5] Charles Perrings, Carlos Castillo-Chavez, Gerardo Chowell, Peter Daszak, Eli P Fenichel, David Finnoff, Richard D Horan, A Marm Kilpatrick, Ann P Kinzig, Nicolai V Kuminoff, et al. Merging economics and epidemiology to improve the prediction and management of infectious disease. *EcoHealth*, 11(4):464–475, 2014.

[6] Toby Wise, Tomislav D Zbozinek, Giorgia Michelini, Cindy C Hagan, and Dean Mobbs. Changes in risk perception and self-reported protective behaviour during the first week of the covid-19 pandemic in the united states. *Royal Society Open Science*, 7(9):200742, 2020.
